# Laboratory evaluation of the effects of sterilizing doses of γ-rays from Caesium-137 source on the daily flight activity and flight performance of *Aedes albopictus* males

**DOI:** 10.1371/journal.pone.0202236

**Published:** 2018-08-14

**Authors:** Cyrille Lebon, Kevin Soupapoule, David A. Wilkinson, Gilbert Le Goff, David Damiens, Louis Clément Gouagna

**Affiliations:** 1 Institut de Recherche pour le Développement (IRD, Unité Mixte de Recherche « Maladies Infectieuses et Vecteurs: Ecologie, Génétique, Evolution et Contrôle » MIVEGEC (IRD 224-CNRS 5290-UM1-UM2), Montpellier, France; 2 IRD La Réunion / GIP CYROI (Recherche Santé Bio-innovation), Sainte-Clotilde, La Réunion Island, France; 3 Université de La Rochelle–UFR Sciences—Département de Biotechnologies, La Rochelle, France; 4 Université de La Réunion, Unité Mixte de Recherche « Processus Infectieux en Milieu Insulaire Tropical (UMR PIMIT)», INSERM U1187-CNRS9192-IRD249. Plateforme de Recherche CYROI, Ste Clotilde, La Réunion Island, France; University of Nevada Reno, UNITED STATES

## Abstract

The control of *Aedes albopictus* through Sterile Male Releases requires that the most competitive males be mass-reared and sterilized usually with gamma- or X-ray radiation prior to release. Developing an understanding of the impact of irradiation treatment on flight performance in sterile males is very important because any fitness cost may reduce the efficacy of SIT intervention in the field. Here, we examined the role of irradiation exposure and sugar-feeding on daily flight activity and performance of *Ae*. *albopictus* males sterilized during pupal stage with gamma-radiation at 35Gray from a Caesium 137 source. We used a previously developed automated video tracking system to monitor the flight activity of different groups of sterile and control non-sterile males over 24 hours in a flight arena. This monitoring took place under controlled laboratory conditions and we wished to quantify the daily flight activity and to highlight any changes due to radiation treatment and nutritional conditions (starved versus sugar fed). Our experimental evidence demonstrated a characteristic diurnal flight activity with a bimodal pattern regardless of the treatment. Precisely, both irradiated and non-irradiated males exhibited two distinct peaks in flight activity in the morning (6–8 a.m.) and late afternoon (4–6 p.m.). Under changing physiological conditions, irradiated males were generally more active over time and flew longer overall distances than control male populations. These results suggest some internal circadian control of the phase relation to the light-dark cycle, with evidence for modification of flight performance by nutritional status. The fact that daily activity patterns are alike in irradiated and control *Ae*. *albopictus* males, and that sterile males could display higher flight performance, is in contrast with the hypothesis that irradiation treatment appears to reduce the fitness of male mosquitoes. We discuss the implications of the present study in sterile-male release programs against *Ae*. *albopictus*.

## Introduction

*Aedes (Stegomyia) albopictus* (Skuse, 1984) (Diptera: Culicidae) plays an important role as a vector of chikungunya, dengue, and other arboviruses, although its vector role is often assumed to be less than that of its sibling, *Aedes aegypti* [1–3]. The limitations of existing strategies based on reduction or removal of mosquito breeding sites often in parallel with the use of broad spectrum insecticides [4,5], have resulted in extensive efforts to develop alternative, eco-friendly control methods against these notorious arbovirus vectors. The Sterile Insect Technique (SIT) is at the front line of such anti-vector campaigns, as it offers a highly selective means of controlling vector populations in synergy with concerns for human health and environmental safety [6, 7]. It is an autocidal method in which mass-reared, irradiated males are released in overwhelming numbers to compete for mates with wild insects [8, 9]. The mating of sterilized insects with wild females produces no viable eggs, leading to significant suppression of wild mosquito populations, especially when several consecutive releases are performed over relatively short timescales. The effectiveness of area-wide implementation of SIT has been demonstrated previously in control of the tsetse fly; vector of *Trypanosoma brucei*, the causative agent of sleeping sickness in humans [10], and in several trials against devastating agricultural and livestock pests [11].

The emergency created by the 2005–2006 chikungunya epidemic outbreak [12] as well as continued burden of disease from chikungunya and the recent outbreak of *Ae*. *albopictus-*spread dengue [13, 14], have established La Reunion Island as a high-risk setting for vector-borne disease emergence and re-emergence. Consequently, the French government has initiated intensive vector surveillance and disease monitoring in the region. Beyond appropriate use of current acceptable insecticides (e.g. deltamethrin) for aerial spraying, new vector control strategies are required to mitigate the threat posed by mosquito-borne diseases on public health. To that end, efforts have been taken to investigate the feasibility of a novel vector control approach that integrates the release of radiation-sterilized males against *Ae*. *albopictus*, and these efforts are currently advancing towards field testing in La Reunion Island. The success of this strategy in mosquito control depends heavily on understanding the biology and the behavior of mosquitoes to calculate the necessary scale of release campaigns and ensure the competitive performance sterile males. In brief, considerable progress has been made in refining the scientific understanding on the biology [15], genetics and phylogeny [16], mating behaviour [17], geographical distribution [18–20], and dispersion [21] of wild mosquito populations. In addition, mating competitiveness studies in the laboratory and semi-field conditions have shown that *Ae*. *albopictus* males sterilized with gamma-radiation at 35Gray during the pupal stage of development could effectively compete with wild males and induce high levels of sterility in females [22, 23].

The key to the effective implementation of SIT approach is a mass production of competitive sterile males that are subsequently released into the wild in overwhelming sterile to wild male ratios. This is to ensure that they take precedence over wild males when mating with females. Lees *et al*. suggested that this requirement can only be achieved if in all biological functions [24], except for fertility, the sterile males remain as competitive as wild males (see for example [25, 26], and if the release ratio of sterile males relative to wild males remains high over several generations. Selective pressures during mass rearing [27], and somatic damages induced by irradiation [28–30] may result in a reduction in the relative fitness of sterile and wild males. However, theoretically, a greatly increased sterile to wild male ratio may compensate for any reduction in the competitiveness of sterile males due to irradiation. A fundamental step for this strategy is the understanding of whether males of the mosquito strain mass-reared in the laboratory and irradiated prior to being release have characteristics of their wild counterparts in terms of sexual behavior and performance. One behavioral trait that we assumed to be an adequate surrogate for competitive fitness of irradiated *Ae*. *albopictus* males, and that is most relevant in the context of the present study is their activity rhythm in response to the environmental factors. Whether sterile males can fly at the appropriate times to ensure mating success in field settings is still unknown, at least in *Ae*. *albopictus*.

In any mosquito species flight is the primary mechanism for dispersal [31], which allows species reproduction as they move either in the search of nutrient-sources which provide optimal fitness acquisition opportunities [32–34], to gain access to mates for males or alternately host seeking and laying eggs for females. Each of these behavioural actions display temporal rhythms [35–37], and different condition dependencies in relation to ecologically relevant life-histories of insects [38, 39]. For instance, there is some evidence that *Aedes* spp. females in different physiological stages exhibit specific daily activity patterns, and adjust their behavioural activities largely in response to variations of some environmental factors [40–43] and associated changes in physiological conditions [43, 44]. The published analyses of flight periodicity of *Ae*. *albopictus* males are scanty, though information on their daily activity pattern and dispersal ability may provide a basis for work on the characteristics of their feeding and mating behaviours in the field. So far, an unresolved issue is whether radiation exposure of male mosquitoes would influence their flight ability and daily activity pattern. Put simply, sterile males will need to show up in the right place at the right time in order to ensure mating success in the field. Any phase shift of activities of irradiated males or any deviation from the ‘norm’ may reduce the probability of encounter between released individuals with their wild congeners, and therefore negatively influence the efficacy of SIT interventions.

Herein we used an automated video tracking system to improve our understanding of the influence of irradiation-induced sterility on life history traits involved in dispersal and mating success. This objective was achieved by comparing the daily activity pattern and flight performance for groups of *Ae*. *albopictus* sterile males and non-sterile males, with and without access to sugar meal under laboratory conditions.

## Materials and methods

### Source insects

This study used samples of *Aedes albopictus* adult males from laboratory colony kept in breeding at the insectary facility located at the CYROI (*Cyclotron Réunion Océan Indien*) technology platform, Saint Denis, La Reunion Island. This colony was initiated in January 2009 from eggs originally collected during field surveys at the north-east of La Reunion Island. The colony is being maintained in the insectary under climate-controlled conditions, at a temperature of 27 ± 2°C, 75 ± 2% relative humidity and a 12L:12D photoperiodic regime. Control of climatic conditions is provided by a humidifier and a thermo-hygrometer for air conditioning. Details of the rearing procedure were as previously described by Damiens *et al*. [23], and specimens used for the experiments were obtained from the 48th and 49th generations of the colony.

Male and female pupae were separated manually within 4 to 12 hr of pupation under a binocular microscope (Leica® MZ6) based on the morphological difference of the genitalia, sufficiently marked in *Ae*. *albopictus* to allow sexing. After sex separation, random batches of males and females from the same larval cohorts were placed separately in different plastic containers. Male pupae were further divided in two groups: one treatment and a control group. Pupae intended for irradiation (treatment) were placed in jars and closed with plastic lids and Parafilm® to avoid water leaks. To produce sterile males, separate batches of 100 pupae each (aged 24–30 h) were sterilized using gamma-radiations from a Caesium-137 source (Gammacell IBL 437, Cis Bio International, Germany). The chosen irradiation dose was 35 Gray (Gy) delivered at 2.35 Gy.min^-1^ for 15 min, a sterilizing condition that was previously shown to induced 95–97% sterility in *Ae*. *albopictus* females when mating with sterilized males, with limited adverse effects on the survival [45], and competitiveness of males [23].

**Fig 1:**
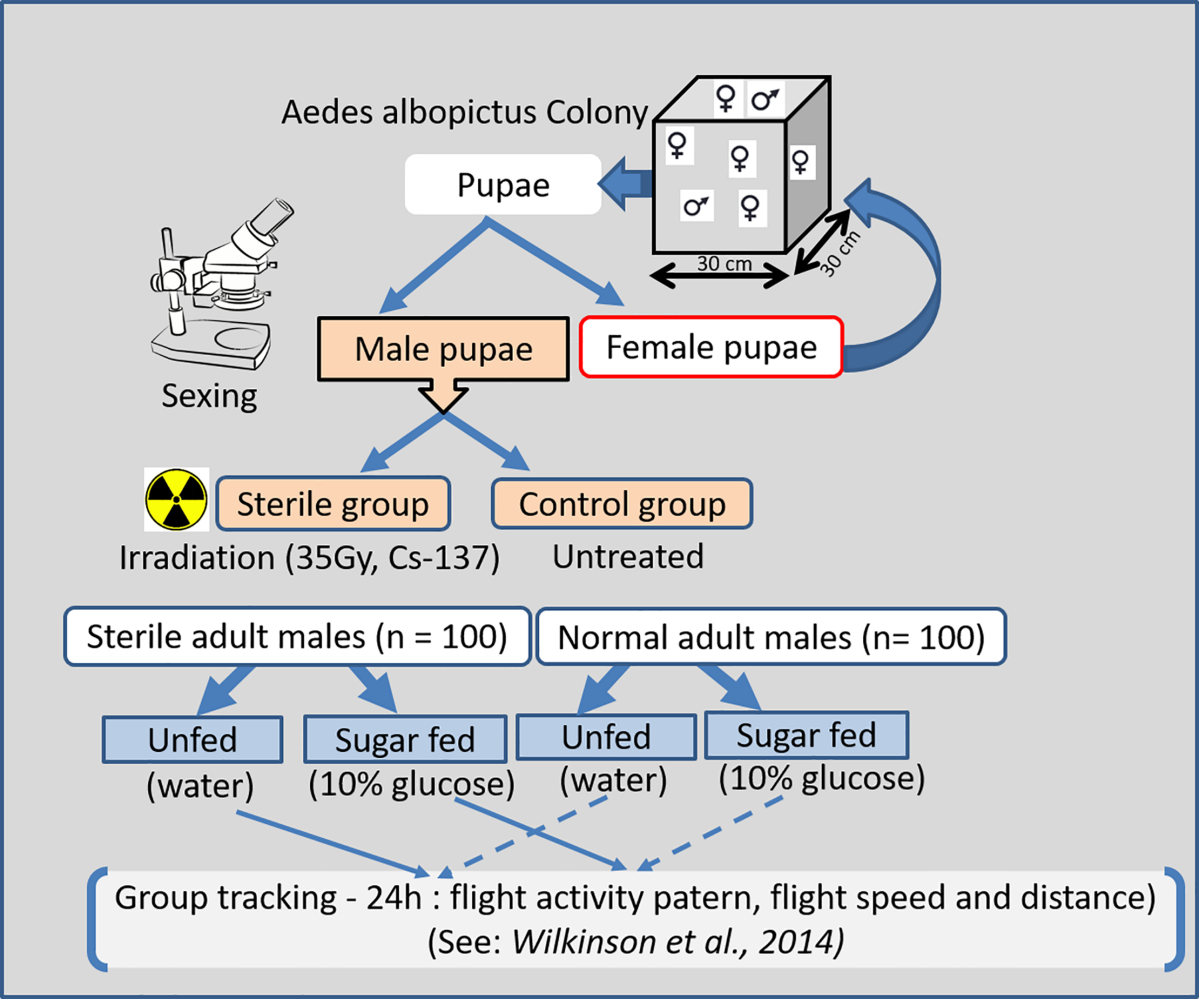
**Schematic representation of the experimental design for evaluating the flight activity of groups of normal males (A) and gamma-irradiated (B) *Aedes albopictus*.** We tested the hypothesis that gamma-radiation treatment of *Ae*. *albopictus* male pupae at the sterilizing dose of 35gy induces changes in flight performance in adult sterile males relative to normal counterparts. Trials using video tracking were performed with groups (n = 15–20) randomly chosen individuals among newly emerged normal (control) and gamma-irradiated group, with varying nutritional status (sugar-fed vs unfed). Following termination of an experimental run, data from the basic records were computed and flight characteristics of each treatment groups and used for statistics.

Subsequently both irradiated pupae and non-irradiated male and female pupae were allowed to emerge in different plastic cages (30 x 30 x 30 cm, Bugdorm, MegaView, Science Education Services Co., Taichung, Taiwan) with continuous access to a 10% (w/v) sucrose solution for 2 days. In this paper "sterile males” and "normal males” refer to male adults that emerged from radiation-treated pupae and untreated pupae, respectively. Immediately after emerged adult males in each treatment were divided into two groups of 15–20 males each: the first was starved, being provided only water from cotton wick soaked in distilled water for 24h, and the second was fed *ad libitum* 10% (w/v) glucose solution from cotton wick for 24h. Thus, on each trial a total of 4 conditions were established (Fig 1): fed or unfed sterile males, fed and unfed normal males. All adult males used for experiments described below were less than 48 hrs old.

### Effect of irradiation on flight performance

To determine the possible influence of irradiation on flight activity over 24h, we used an automated video tracking system similar to the type described previously [46] to track the locomotion activity of different groups of sterile and normal *Ae*. *albopictus* males of two nutritional treatments (starved and sugar fed). The logistics of such a system consists of two separate flight arenas (30 x 40 x 10 cm in size each) allowing groups of mosquitoes to flight spontaneously, and at the same time a coupled motion-capture video system enables real-time tracking in both day and night-time under laboratory environments. Simulation of flight activity of multiple mosquitoes from 2D video images has previously shown that estimates of average distance, time, and flight speed from such a system have a minimum accuracy of 90%. Further details of the multi-target tracking algorithm employed and the implementations of the system are presented elsewhere [46].

Tracking the daily activities of sterile males and normal males was performed with identical experimental setups. For practical reasons, measurement of flight activity could only be started during working period: tracking usually began around 12:00 a.m. on the first day, and was stopped at 18:00 on the second day. The 24h-period over which males were subjected to the activity monitoring encompassed both the scotophase and the photophase. Two replicates of such behavioural assays were conducted at intervals of 4 days during which the flight activity of random batches (n = 15–20 males each) of starved and freshly fed from the sterile or non-sterile groups, respectively, were placed in individual arenas, 30 minutes before the start of filming to allow an adjustment time to the laboratory conditions.

### Evaluation of flight activity and performance of sterile versus normal males

As validated recently [46], the video images of the flight tracking were recorded at 25 frames per second, with a resolution of 1024 * 768 onto a portable external hard drive. The data were processed in MATLAB R2012b, run under a 64-bit Windows 7 Professional environment on an Intel i7-2600 (3.4GHz four-core processors—16 Gb (4 * 4 Gb)) of DDR3 RAM. From each record, centroid positions of each mosquito were defined after subtraction of a background image with the aid of a custom-programmed graphical user interface. A flight speed histogram was generated from data collected and allows logical separation of movement activities into stationary and mobile categories. Mosquitoes walking behavior were not considered in this study, and any mosquito at a speed of ≥ 5 cm/s was considered to be in movement. Average activity was considered across all mosquitoes from all experimental repeats, and the same velocity cut-off value was use for all data. Temporal trends in these data provided information on the periodicity of flight activities, i.e. episodes of continuous flights or erratic flight impulses and periods of rest for each batch of mosquitoes tested. In addition to these three types of activities defined based on the speed of movement, the flight performance for sterile and normal males under various nutritional treatments was assessed, taking into account the total distance flown, calculated by adding all the flight episodes by individual males during the first 24 hours of the trial time.

### Statistical analyses

The data in each experiment consist of a video tracing activity of groups of sterile and normal male mosquitoes over 24-hours in the experimental arena. Computer assisted tracking algorithms allowed calculation of three variables: average percentage of mosquito flight time per hour (ie. the proportion of total possible time that mosquitoes spent in flight), average total distance flown (in meters, calculated as the sum of all distances travelled divided by the number of mosquitoes), and average flying speed (meters per second). Data recorded from 2 different replicates were used for statistical analysis using SPSS software (IBM SPSS package vers.23 for Windows). Throughout the manuscript means are reported ± Standard Error of Mean. The temporal activity pattern was visually examined by depicting the proportion of mosquitoes in-flight over time. Univariate General Linear models (GLM) were performed on pooled data to disentangle the effects of irradiation treatment (yes = 1, no = 0), nutritional condition (sugar fed = 1, unfed = 0), and their interactions on the flight activity (i.e. the proportion of active males), and performance parameters (flying speed and distance flown). Data recorded in all experiments were normalized by logarithmic (Log_10_) transformation prior to analyses (S1 Dataset). Significant GLM analysis was followed by Tukey post hoc contrasts to differentiate the nature of statistical differences between group means. The statistical test for all general linear models analyses was the F-value, and the significance level (α) for all statistical significant tests was set at 0.05.

## Results

### Effect of irradiation on flight activity and diel flight pattern *of Ae*. *albopictus* males

**Fig 2:**
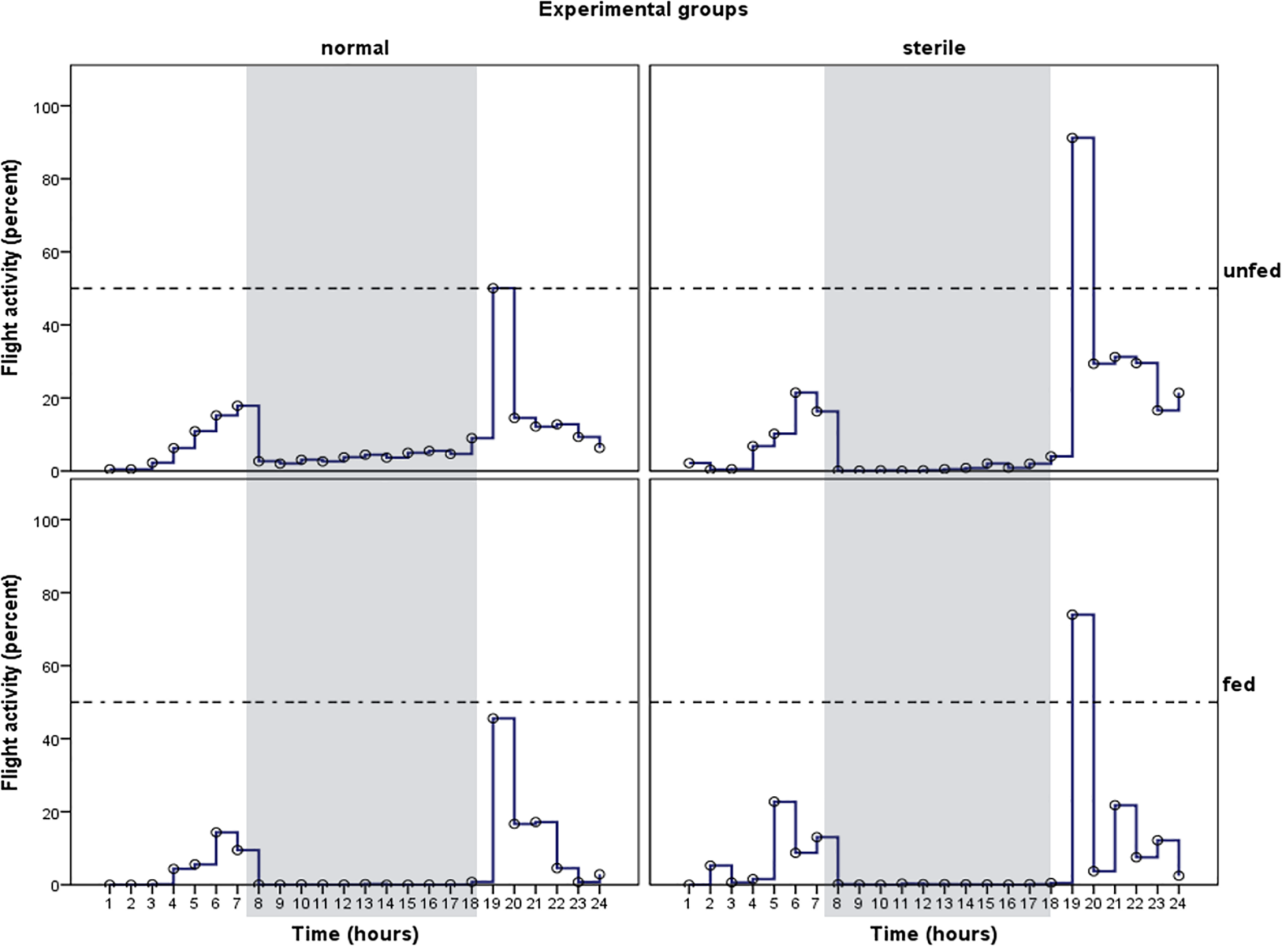
**Daily flight activity rhythm of groups of normal males (A) and gamma-irradiated (B) *Ae*. *albopictus* in a flight arena**. Each line indicates the occurrence of flight activity, i.e. the mean number of active males relative to the total number of individuals present, within each treatment group. The scotophase is indicated by a vertical grey bars. Recording was performed using single calibrated CCD camera that tracked flight paths at a frequency of 60 frames per second for group of 15–20 males released in a 25 x 25 cm flight arena established in a laboratory- controlled constant regime of 12L: 12D. Multi-object tracking algorithms on standard computational hardware was performed with dedicated software, as previously described in Wilkinson *et al*. (2014). The experiments were normally continued for a 24h period (x 2 replicates) and the number of active male were averaged to produce the distribution of activity against time for each treatment group. For graphical visualization of the data, means from the untransformed data and their SEMs were used. Note the different magnitude of the flight activity for the different group.

Measurements of the 24-hrs cycles of flight activity were generated from 8 videos independently recorded from different paired experiments using groups of sterile and normal *Ae*. *albopictus* males, either under fed or unfed conditions (n = 15–20 males mosquitoes in each of the 4 conditions x 2 replicates). Over the 24h experimental period the observed number of active males varied independently of the treatment tested, reflecting a random participation of most experimental males in flight activity. Consistently in all treatment groups and across repeats within each group, the temporal distribution of flight activity showed a bimodal distribution pattern that mirrored a regular 24 h circadian rhythm (Fig 2). The hourly activity patterns were stable across replicates. Flight activity gradually increased in the first 6 hours of the experiments, peaking 2–3 h prior to the day-night transition (16h00–18h00). Thereafter, the number of males in flight in each group was consistently reduced (or zero) during the night time and the most part of the photophase (from 7h00 to 17h00 under our experimental conditions). Subsequently, the individual males in each group showed a distinct increase in activity, reaching a second peak around 18h00-19h00, and gradually decreasing following the onset of scotophase. Overall, results indicate that sterile *Ae*. *albopictus* males under both unfed and sugar-fed conditions showed diurnal flight pattern and synchronized their activity cycle in a manner that is most expected from normal males (Fig 2).

The effect of time on flight activity during the 24-hrs of total video recording time was statistically significant (*F*_*23*,*165*_ = 7.11, *p* < 0.001), with both sterile and normal males being more active at 06h00–08h00 and 16h00–18h00. During these phases of the activity cycle, the number of flying individuals observed among unfed males was greater than that obtained from sugar-fed ones (Fig 2), and this tendency was statistically significant both in the control (*F*_*1*, *95*_ = 19.52, *p* < 0.001) and irradiation-treated (*F*_*1*, *95*_ = 6.09, *p* = 0.01) groups. This observation indicates that besides the predictable temporal activity pattern over the daily cycle, changes in flight behaviour of *Ae*. *albopictus* males is also state-dependent, with a highest flight activity being observed in unfed males probably in search of sugar-source, and an apparent decrease of flight activity during the ingestion period.

The distribution of flying males during a 24-hr period of observation was the similar across the sterile and control groups, indicating no adverse effect of irradiation upon flight activity of *Ae*. *albopictus* males. In fact, the relative magnitudes of activity peaks were higher in sterile than normal males (Fig 2), but differences in the hourly average proportions of males in flight were not statistically significant, either for unfed (*p* = 0.36) or fed groups (*p =* 0.39). In addition, the control unfed males seemed to forage more during the night than the sterile males of the same nutritional status (Fig 2), although this night-time activity was more related to walking behaviour than flying behaviour. Overall, controlling for the effect of time, the distribution of flights for a male mosquitoes was only significantly affected by nutritional status (GLM, *F*_*1*,*165*_ = 39.30; *p* < 0.001). In contrast, the irradiation treatment (*p* = 0.41), or the interaction of irradiation and nutrition (*p* = 0.19) did not influence the flight activity significantly (Table 1).

**Table 1:** Independent predictors of daily flight activity and performance of normal and gamma-radiated *Aedes albopictus* males. Results of Generalised Linear Models (GLM) on the number of active mosquitoes, flight distance and flying speed of groups of *Ae*. *albopictus* males. In each univariate model, independent variables were added to the model if p < 0.05 (adjusting for time in the 24hrs cycle).

Source of variation Outcome		Type III Sum of Squares	Degree of freedom	Mean Square	F-test^a^	p-value^b^
**Intercept**	Flight activity	74.85	(1, 165)	74.851	142.699	<0.001
	Flying speed	144.94	=	144.944	2699.69	<0.001
	Flight distance	116.27	=	116.274	40.78	<0.001
**Group**	Flight activity	0.001	(1, 165)	0.001	0.012	0.91
	Flying speed	0.16	=	0.160	7.36	0.007
	Flight distance	0.004	=	.004	0.01	0.892
**Status**	Flight activity	2.89	(1, 165)	2.897	39.30	<0.001
	Flying speed	0.00	=	0.000	0.01	0.91
	Flight distance	10.768	=	10.768	51.80	<0.001
Group * Status	Flight activity	0.123	(1, 165)	0.123	1.67	0.19
	Flying speed	0.01	=	0.017	0.78	0.37
	Flight distance	0.61	=	0.614	2.95	0.08
Time of the day	Flight activity	12.06	(23, 165)	0.525	7.11	<0.001
	Flying speed	1.27	=	0.055	2.55	<0.001
	Flight distance	65.57	=	2.851	13.71	<0.001

a. The F tests the effect of Group based on the linearly independent pairwise comparisons among the estimated means

b. Two-tailed tests. P values were corrected for multiple testing (eight tests) using the false discovery rate. The mean difference is significant at the 0.05 level.

### Effect of irradiation on flight performance

**Fig 3:**
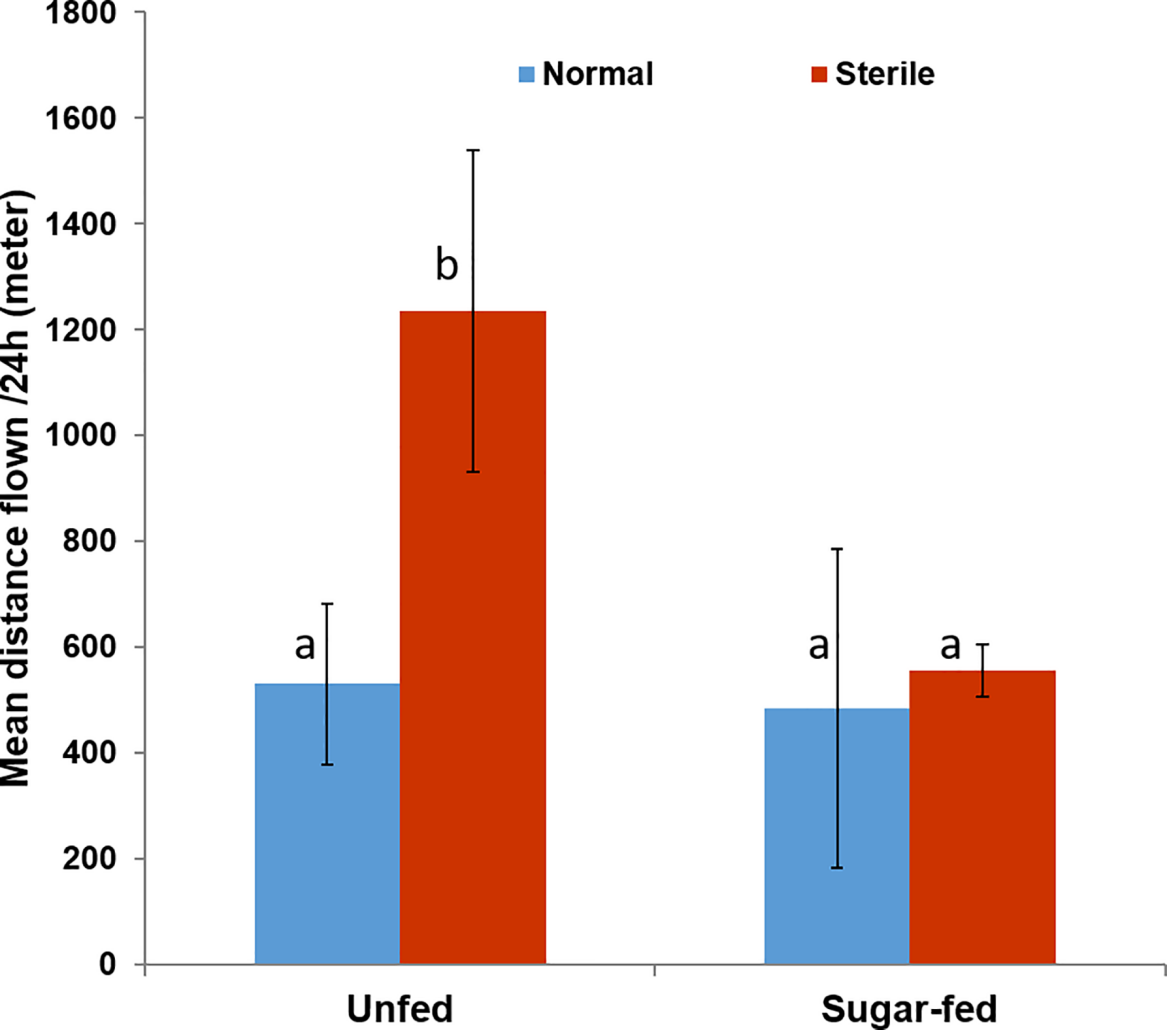
Comparison of flying speed of active *Aedes albopictus* adult males in respect to irradiation treatment and nutritional status. The experimental period of 24h was considered for determining the mean flight velocity by individual groups of mosquitoes. Bars are the mean values obtained from groups of individuals (irradiated and control) assayed both when they had fed sugar solution and water after emergence. Error bars give the binomial standard error. The effect of irradiation and nutritional was measured using generalized linear models (GLM, α = 0.05) on the log-transformed data. Bars with the same letter indicate that the results are not significantly.

Fig 3 depicts the distributions of the distances flown over 24h for sterile and normal *Ae*. *albopictus* males, either with or without an initial sugar meal. The mean distance flown during the experimental period was significantly greater in unfed than in sugar fed males, both in the sterile and control groups (Fig 3). For instance, unfed sterile males were observed to move on the longest distance (1348 ± 268 meters) compared to sugar-fed ones (650.3 ± 276 meters) (F_1,143_ = 4.09, *p* = 0.037). Similarly in the control group on the other hand, the different in mean flight distances between the sugar-fed (553 ± 262 meters) and unfed males (596.4 ± 283 meters) was not statistically significant (*p* = 0.88). Further statistical analyses showed that over the course of 24-hrs, irradiated males tended to fly longer distances than the control group, but the difference was not statistically significant, both for unfed (*p* = 0.47) and sugar-fed males (*p* = 0.07). Controlling for the confounding effect of time (which effect on the distribution of flight activity was statistically significant in all group (Table 1), the subsequent full GLM analyses confirmed the variations in mean flight distances was significantly affected by nutritional status (*F*_*1*,*165*_ = 51.80, *p* < 0.001), but not irradiation treatment (*p* = 0.89), with a non-significant interaction term (*p* = 0.08).

**Fig 4:**
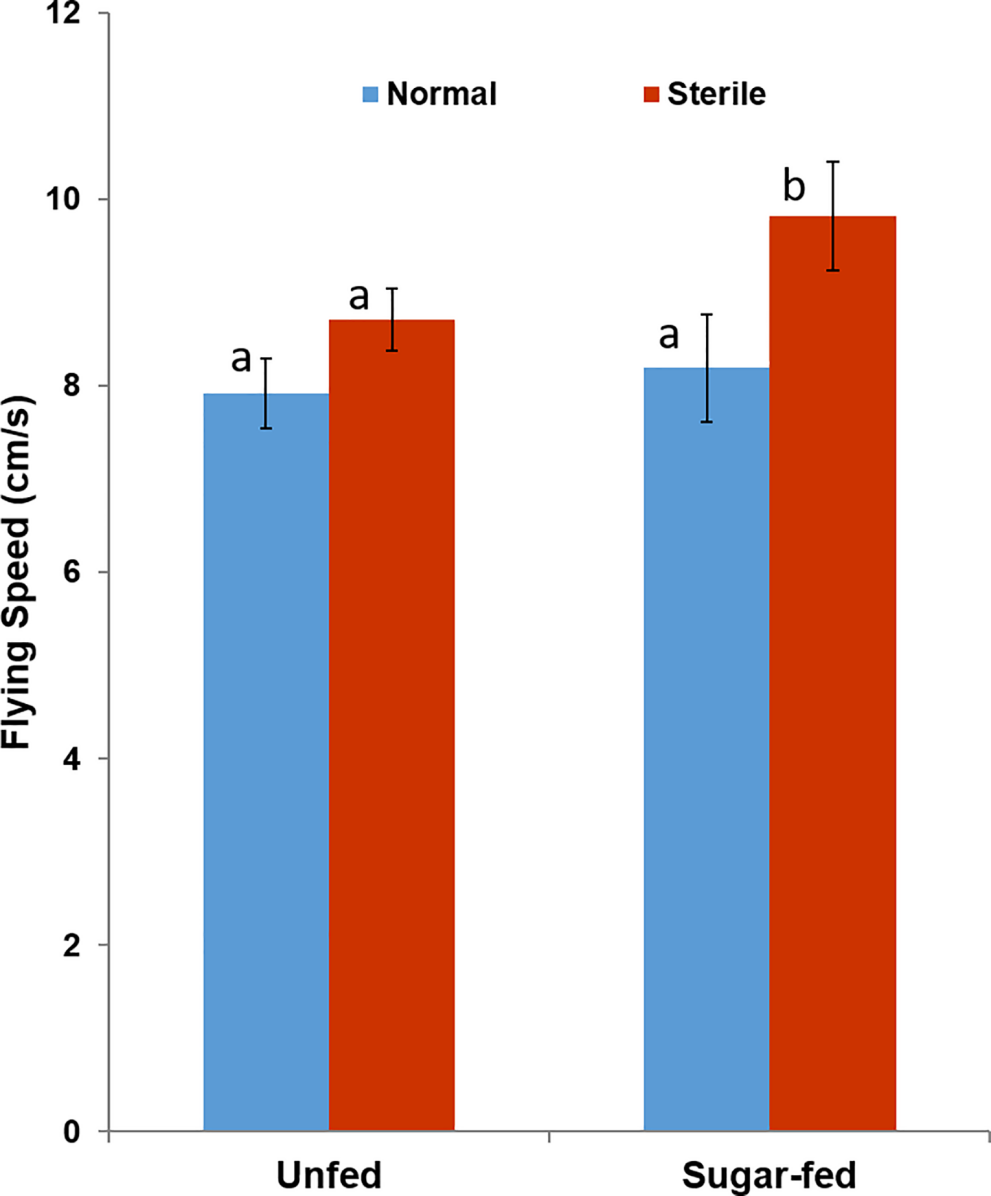
Effects of irradiation treatment and nutritional status on the average distance flown by active *Aedes albopictus* adult males. Experiments were conducted in duplicate for 24 h on the flight arena at 28°Cand 70% RH, with 12D:12L photoperiod. ** Each bar represents average of the untransformed data. Error bars give the binomial standard error of the mean. The effect of irradiation and nutritional was tested using generalized linear models on the log-transformed data. Bars with the same letter indicate that the results are not significantly different. Difference is significantly at α = 0.05 in Tukey’s HSD post-hoc test following a univariate GLM procedure.

In reference to flight velocity during the time dedicated to activity (Fig 4), pairwise comparisons showed no significant differences between sugar-fed and unfed males (*p >* 0.01), across the treatment groups. Indeed, in the control group, the mean flying speed was comparable between unfed (Mean ± S.E.M.: 7.91 ± 0.37cm*S^-1^) and sugar-fed individuals (8.19 ± 0.57 cm*S^-1^) (*p = 0*.*66*). In the irradiation treated group, mean flying speed for unfed males (8.70 ± 0.32 cm*S^-1^) and sugar-fed (9.81 ± 0.5 cm*S^-1^,) sterile males was also similar (*p* = 0.46). The full statistical model showed that whereas male flight velocity changed over time (*F*_*23*,*143*_ = 2.55, *p* < 0.001), there was no significant effect of either irradiation treatment (*p* = 0.07), or nutritional condition (*p* = 0.91), with also a non-significant irradiation by nutrition interaction (*p* = 0.37) on flying speed.

## Discussion

It has generally been established that ionizing radiation generates chromosomal aberrations and dominant lethal mutations in sperm [29], but somatic damage induced by irradiation may reduce the competitive fitness of sterile males, even in the case of low-level radiation such as that applied in the present study. The specific irradiation dose used here (35 Gy) has been experimentally optimised to achieve maximum dose-sterility curve while minimising deleterious high-dose effects on the overall fitness of males [47]. In addition, initial mating experiments with irradiated males provided assurance that gamma-irradiation treatment at 35Gy achieved the specified requirement for sterility [23]. Herein, we attempted to determine what effects, if any, irradiation treatments and nutritional conditions would have on the daily activity patterns and flight performance of *Ae*. *albopictus* males, with possible consequences for their performance in SIT campaigns.

Our results showed that the daily activity patterns of sterile and normal males are similar, being nearly diurnal with consistent bimodal circadian rhythm that comprises one activity peak in the dawn (6:00–8:00 a.m.) and another peak in the afternoon (4:00–6:00 p.m.). Interestingly, this observation confirms previous results reported in Wilkinson *et al*. [46]. In addition, despite the physiological differences in both cohorts, the daily rhythms of activity in sugar-fed and unfed males was synchronous, yet at the onset of activity peaks, the amplitude of flight activity was clearly increased in starved sterile males relative to sugar-fed males. This suggests that nutrition was more important than irradiation in influencing the level of flight performance. Several reasons could explain the stability of the daily activity pattern in sterile and normal males.

The observation of a clear and reproducible periodicity of flight activity in sterile and normal *Ae*. *albopictus* males confirms previous report of a temporal stability of bimodal activity pattern of local of *Ae*. *albopictus* strains with the morning and late afternoon peaks, both in field population [48] and in laboratory setting [49]. Our experimental system did not classify male behaviour with respect to a set of behavioral interactions, but rather it detects and quantifies male flight activity in terms of the number of active (flying) males over 24hrs. Other studies examining mating and sugar feeding patterns of *Aedes* spp., demonstrated a diel pattern in both males and females [50–53], with a small peak in the morning and a large peak in the evening. Evidently, mosquitoes have evolved mechanisms to schedule their movement, feeding and mating to the optimal time of day, punctuated by intervals of inactivity or rest. Based on the knowledge of the biological rhythms of mosquitoes [1, 40, 43, 54–56], the observed temporal periodicity in *Ae*. *albopictus* flight activity may be controlled by at least three types mechanisms: (i) exogenous stimuli such as environmental conditions (i.e. ambient light, UV radiation, temperature, humidity, and resource availability) which have predictable 24 hour cycles and (ii) periodically controlled response mechanisms, with a specific response relative to the perception of specific stimuli, and (iii) endogenous self-sustaining mechanisms that are persistent in the absence of any external signal. Details of the endogenous circadian clock underlying daily behavioral rhythms in major disease-vector species have been described previously [56, 57–59]. The lack of divergence in flight activity periods between irradiated and normal *Ae*. *albopictus* males is an indication that endogenous circadian rhythms controlling flight activity periodicities was not altered by irradiation treatment. Perhaps, reproducing the experiments for longer time spans with regular sampling might be required to improve our understanding of the daily activity of sterile males and the reproducibility of the oscillation phase of feeding and mating behaviours. However, our results challenge the generality of the claim that irradiation negatively impacts the vitality and competitive mating ability of sterile insects relative to wild types [60, 61]. The potential debilitating effects of irradiation-treatment had motivated the exploration of genetic control alternatives to irradiation [62, 63].

Changes in the flight activity and ability to disperse are of great importance in the application of radiation for insect control programs. For this reason, early research on mosquito radiation treatments has been designed to induce an adequate sterility level without causing loss in the overall fitness [22, 23, 64–66]. The observation that unfed *Ae*. *albopictus* males exhibit more activity displays than fed individuals may be related to the search for sugar source and decreases during the ingestion period in recently fed males. The observation that unfed *Ae*. *albopictus* males exhibit increased activity when unfed is likely the result of an increase in foraging behaviour. Interestingly, control males demonstrated some movement at night-time when unfed, suggesting that some foraging activity takes place in those hours that males are usually considered to be at rest. This was less obvious in sterile males, indicating that while the diurnal dispersal cycles were not interrupted by somatic damage induced by radiation, some behaviours may have been. This coupled with the observation that sterilized *Ae*. *albopictus* males flew faster than their control-group counterparts may indicate some changes to the mosquitoes’ metabolisms. Nevertheless, the overall long-term effects of these potential physiological changes on sterile male performance have yet to be assessed.

There is no controversy on the fact that male mosquitoes need an adequate supply of energy for their activity [44, 67–69]. In order to fuel these energy demands, sterile males released in the field would require a constant and sufficiently high supply of sugar, exclusively from plants derived carbohydrates—in fruit, honeydew, floral and extrafloral nectar [70, 71], a prerequisite apparently easily achieved by wild *Aedes* mosquitoes, considering the high rate of plant sugar consumption in field [72–75],. Indeed, wild *Ae*. *albopictus* will obviously get many of these energy reserves from plants, on which they feed intermittently through daily activity [76, 77], during which time they frequently alternate between energetically costly flight and rest. As the results of the present study suggest, the unfed sterile males are likely to increase their activity in cases of sudden stress affecting the physiological fitness in the field situation, where they need to find sugar resources and mates for sexual reproduction.

In conclusion, despite the simplified experimental conditions of the presented study (i.e. a low spatial resolution in flight arena) relative to an in-field situation, our important finding is that irradiation treatment does not seem to significantly hinder parameters of flight (flight speed, or distance flown) that may impact competitiveness, at least under our experimental conditions. More importantly, we showed for the first time that sterile males are able to match the activity patterns of wild males, an outcome that is highly important for SIT success. Furthermore, the flight activity observed in unfed sterile males suggests that approximately normal foraging activity is likely in field scenarios. However, recent studies have demonstrated that a pre-release provision of nutritional supplements to sterile males shortly after their emergence, along with optimizing holding conditions [23, 46, 78] could provide better fitness and optimize subsequent survival and reproduction in the open field. In addition, un-modified temporal trends in flight activity between sterile and control males suggest that these populations are likely to interact at the correct times of day following release. Examining such temporal variations in mating and sugar feeding rhythms and daily frequencies of *Ae*. *albopictus* populations is challenging under field conditions due to accessibility, lighting conditions and mosquito population dispersal. Further research directed at comparing the daily behavioural rhythms in irradiated vs. normal male mosquitoes, and their dispersal capacity and rate of fuel utilization under field conditions would enhance our ability improve sterile male competition upon release into open fields.

## Supporting information

S1 Dataset (XLSX)
